# Evaluating Total Mercury and Methyl Mercury Contents in Canned Tuna Fish of the Persian Gulf

**Published:** 2018

**Authors:** Ali Bashiri Dezfouli, Jamileh Salar-Amoli, Tahereh Ali-Esfahani, Hedayat Hosseini, Kiandokht Ghanati

**Affiliations:** a *Department of Basic Sciences, Faculty of Veterinary Medicine, University of Tehran, Tehran, Iran. *; b *Toxicology and Animal Poisoning Research Center, Faculty of Veterinary Medicine, University of Tehran, Tehran, Iran. *; c *National Nutrition Food Technology Research Institute (NNFTRI), Shahid Beheshti University of Medical Sciences, Tehran, Iran. *; d *Food Safety Research Center, Shahid Beheshti University of Medical Sciences, Tehran, Iran.*

**Keywords:** Mercury, Methyl mercury, Canned tuna, Persian Gulf, Food hygiene

## Abstract

Due to hygienic risks of mercury residues in food and marine originated supplements, measuring total mercury and methyl mercury contents of canned tuna as a highly consumable marine food product is essential. In this study, 40 canned Tuna fish (from Persian Gulf) were collected in 2015 and then flame atomic absorption spectrometer (FAAS) and thermo gas chromatography mass spectrophotometry were used to measure total mercury and methyl mercury, respectively. The results indicated that the average contents of total mercury and methyl mercury of the canned tunas, with 34.2 and 29.5 ppb decrements compared with 2009’s measurement, were 177.4 and 143.7 ppb respectively. The highest concentration of the total mercury was 315.2 while it was 267.9 ppb for methyl mercury. This study showed that the content of the mercury in canned tunas of the Persian Gulf was less than the Maximum Residue Limit (MRL).

## Introduction

Mercury is one of natural heavy metals that could cause food-borne toxicities by contaminating different nutritional levels. This element is also an environmental pollutant due to the stability and the ability to accumulate in biologic tissues (1). Mercury is foundin natural sources such as mines and mountains and released into the environment after industrial activities and climate changes. Both natural processes such as soil erosion and volcano eruption and human activities such as electricity generation, steelmaking, fossil fuels and residue discard play roles in releasing this element to the environment (2).

Mercury usuallyis released into the environment in the form of inorganic and some aquatic microorganisms causemercury methylation and change its form to the organic one, methyl mercury (3). Mercury attaches to thiol group of the cysteine available in proteins of various fish body parts and this causes not getting destroyed even after preparation and cook (4). According to the results, methyl mercury constitutes 90% of the total mercury in tuna fish (5, 6). Although people could receive mercury from foods, drinks and even air (7), eating contaminated fish is the most important cause of mercury poisoning according to the US Environmental Protection Agency report (8, 9).

Aquatic food products provide a significant part of daily food. Hence, receiving methyl mercury through fish consumption could threaten the health of the population. The level of mercury accumulation in different parts of the fish body depends on the age, place and feeding. Therefore, large species of fish have the highest concentration of the mercury in various organs as the last part of the food chain (10, 11). The accumulation in large fish species is so high that several studies indicated the relationship between the increasing level of tuna fish consumption and high concentration of methyl mercury in the blood (12). Mercury could easily pass through the placenta causing lesions in the nervous system, behavioral disorders, growth retardation of the embryo and so forth (13-15). Methyl mercury poisoning in adults could impose toxic effects by effecting on cardiovascular system (16, 17). The signs of this poisoning in adults may include ataxia, confusion, unconsciousness and death (18).

Measuring the mercury content is essential based on public health aspect. According to EU announcement, the safe level of mercury in fish is 1 ppm (19). The US Environmental Protection Agency has set 0.5 ppm and 0.7 µg/kg as traces for mercury and methyl mercury, respectively (20). Based on Provisional Tolerance Weekly Intake set by JECFA (Joint FAO/WHO), PTWI is 5 for mercury and 1.6 µg/kg b.w. for methyl mercury (21).

Tuna fish has been consumedwidely all around the world. Mercury poisoning threat is so high that some countries measure the mercury content of the environment and foods continuously or intermittently. The USA analyzed the mercury content of canned tuna in 1998-2003 period and announced that its content has been increased a little since 1991 but it is still less than the trace announced by the FDA (1 ppm) (8, 22). The contents of total mercury and methyl mercury were also measured in canned tuna in a periodic study during 2009 in Iran and the results indicated that the mercury content of the samples was less than the standards (20). Therefore, evaluation of total mercury and methyl mercury contents of various brands of canned tuna fish was conducted in this study in 2015 and the contents were compared with studies conducted previous years.

## Experimental

*Materials and reagents*

 All materials used in this study except mercury standard solution (1000 ppm, Merck Company) and methyl mercury chloride test solution (10 ppm, AccuStandard Company) were analytical grade. All laboratory glass wares were put in nitric oxide 10% for 24 h and rinsed with deionized distilled water prior the usage to prevent any mercury contamination. 

*Sampling*

 Forty canned Tuna fish of 10 different companies were collected randomly in 2015 to measure the total mercury and methyl mercury contents in 2015. The origin of all tuna fish was the Persian Gulf. The samples were labeled in the Toxicology Research Center of University of Tehran and were then kept in a clean and dry location until the examination.

*Preparation and digestion of the samples*

 To measure the total mercury, optimizing of the method and oil isolation of the samples were performed. One gram of homogenized sample were mixed and kept with 5 mL concentrated sulfuric acid and 2 mL concentrated nitric acid for 15 min in the room temperature. Then, the samples were transferred and kept in 90 °C temperature for 1 h. 20 µL permanganate sodium 5% was added to the samples and again they were put in 90 °C bath for complete mercury reduction. 7 mL hydroxyl ammonium 10% had then been added and the samples were filtered. Eventually, the samples were achieved to the required volume with distilled water (23).

 To measure the organic mercury, 1 gram of the tissue was hydrolyzed with 10 mLNaOH 10% for 2 h at 60 °C and then methyl mercury was extracted using a buffer, acetate ammonium solvents and reagents, sodium tetraphenylborate and hexane (24).

*Chemical analysis*

A GBC HG 3000 continuous-flow vapor system equipped with a gas-liquid separator was used for Hg generation.Determination of total mercury performed with a GBC 906 AA Flame Atomic Absorption Spectrometer (FAAS). A mercury concentrator cell was used to perform the analysis. A mercury hallow cathode lamp was used as a light source at 273.7 nm. The method involved the continuous generation of mercury vapor from aqueous sample acidified with HCl to final concentration of 2 M, which were mixed with reducing agent (10% SnCl_2 _solution and 3 M HCl). The mercury was purged from the sample using argon to the mercury concentrator cell and after 45 sec, absorbance of mercury was determined (10, 25).

A thermos gas chromatography-mass spectrometer (GC-MS) was used to determine methyl mercury concentration in samples. Spectrometer equipped with quadruple mass analyzer and electron impact ionization source (70 eV) was used. Interface temperature was set at 280 °C, while mass scan range wasused between 40 and 450 amu. The GC-MS equipped with CP-Sil 5CB (100% polydimethylsiloxane) fused-silica capillary column (25 m × 0.32 mm ID and 1.2 µm film thickness). Operation conditions were as follow: split ratio = 3, helium carrier gas (1.4 mL/min), injector and detector temperature 280 °C and 300 °C, respectively and temperature program: 80 °C (1 min), 320 °C (10 °C/min, 5 min), hydrogen flow rate (30 mL/min), air flow rate (300 mL/min) (20).

*Statistical analysis*

All data are presented as mean values ± SD derived from three independent experiments. Statistical analysis was performed with SPSS software (Version 22.0). The mean concentration of mercury and methyl mercury in canned tuna fish were compared by one-way ANOVA. A *p*-value less than 0.05 was considered statistically significant.

## Results

The precision and accuracy of the method was tested using repetitive spike of a sample and finally, the appropriate recovery was confirmed (Table 1). The limit of detection (LOD) of mercury and methyl mercury are 11 and 6 ppb for fish samples, respectively.

**Figure 1:**
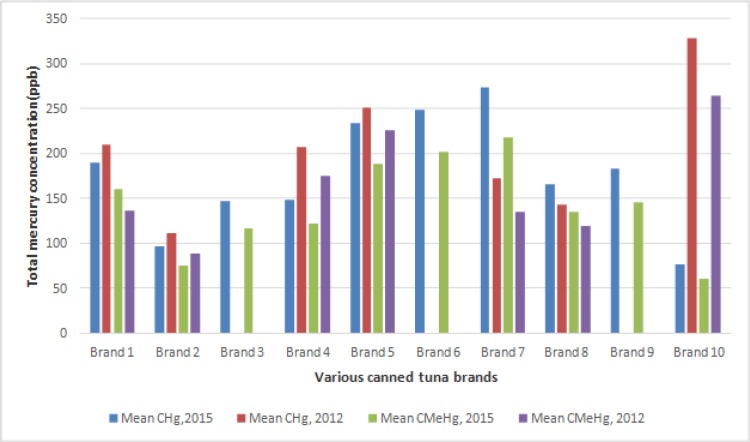
The average of mercury and methyl mercury concentrations of various brands of canned tunas of the Persian Gulf in 2009 and 2015

What is studied in Figure 1 is the comparison of mercury and methyl mercury concentration in ten brands of canned tuna fish in 2009 and 2015. The data showed a significant decrease in the concentration of mercury in five brands. In contrast, it increased in two brands. However, in the case of methyl mercury, only four brands experienced a considerable decrease in 2015 than 2009. It is important to note that three brands analyzed in 2015, were not produced in 2009.

Mercury and methyl mercury concentrations are indicated in Table 2. The results indicated that the range of mercury residues was from 73.6 to 311.22 ppb, with an average of 177.5 ppb. In case of methyl mercury, the range was from 57.5 to 267.9 ppb, with an average of 143.7 ppb. In 7.5% of the samples, the total mercury content was higher than 300 ppb. Also, the percentage of methyl mercury to mercury (MeHg/Hg%) is indicated in Table 2 with the highest (84.7%) and the lowest (77.8%) belonging to Brand 1 and Brand 2, respectively. The results were on the same way with the results of other studies conducted on aquatics. In a study conducted on canned tuna of the Persian Gulf by The Toxicology Center of University of Tehran in 2009, the average concentrations of mercury and methyl mercury were 211.6 and 173 ppb, respectively (Table 3) (20). It was indicated by comparing the results that the mercury concentration of the canned tuna of the Persian Gulf had an increasing trend from 2004 to 2012. However, the trend had a little decrease during the last three years.

## Discussion

 Significant growth of industrial affairs and not following the ecologic principles in discarding the wastes have been exposed the aquatic environment and their products (which was obtained from these origin) to be contaminated with toxic materials. Concerns of Mercury contaminations have been noticed in the Persian Gulf. The involved reasons can be attributed to secondary contamination due to petrochemical activity. Species of Tuna fish (*Thunnus *spp.) and other large fish species have naturally high concentrations of mercury due to bioaccumulation and their position in the food chain (26). The diet could affect the mercury concentration of tuna fish species. Meanwhile, these species swim while their mouths are open and therefore, the solved mercury could easily be absorbed through high-pressure flow of water in the gills causing its concentration to be increased with aging (1, 9). The results of a study indicated a direct relationship between the age, size and mercury content of fish (27, 28). Also, the location of fishing greatly affects the level of mercury accumulation and causes a significant difference among the aquatics fishes in different locations (5). However, the location of fishing is not proposed by the companies to the customers and this makes the spatial comparison difficult. Many advantages have been mentioned for consuming sea foods by the pregnant and kids. Tuna fish is a good source of vitamin E, protein and essential fatty acids (6).

 Meanwhile, this commercial product could be easily and numerously provided. Canned tuna is consumed by approximately 80% of the Iranian (32% fewer than once a month, 28% once a month, 25% three-four times a month, 4% more than four times a month) (20). Since the level of accumulated mercury in tuna fish is more than other fish species, some alerts were announced about its consumption during the last decade (29). US Food and Drug Administration (FDA) and US Environmental Protection Agency (EPA) warned about the consumption of this species and its byproducts to endangered population (8, 22). The dangers of the mercury are so high that world organizations recommend to control and to filter it, especially in some food products such as tuna and other canned products. Compared to studies conducted by Toxicology Research Center in the previous years (Table 3), the average and range of total mercury have been decreased. According to the increment of the wastes contained in this element into the environment and releasing mercury to the water sources, the above-mentioned decrement was not expected. This decrement might be due to the location of fishing since mercury concentration of the aquatic animals depends on the mercury content of the surroundings. This content might be affected by industrial activities and water flow and as a consequence, the level of mercury might be changed in tuna fish (30, 31). Since the locations of fishing are secret for the companies and consumers could not get the information about, spatial and distance comparisons of them are difficult. Another possible reason for the decrement in mercury content might be unlimited fishing of tuna fish in which smaller and younger fish is caught in a specific time period and as a results, the accumulated mercury content in tissues is decreased (32). Other possible reasons which should be taken into account are fish size, migration, diets and the level of food consumption by different species. 

 The results of the study indicated that methyl mercury constitutes high percentage of total mercury (80.6%) in canned tuna. The results of the previous studies conducted on tuna fish samples of the Persian Gulf in 2009 reported its level to 80% (20). In another study, a range of 75-100% with an average of 91% was reported for tuna fish (6). This average was higher comparing with 89% average achieved in another study (5). There was no significant difference in mercury content of 10 different brands (*P*˂0.05). The expiration date, packaging environment, price of the product and the production season were all ineffective on mercury content. Five, three and two brands of the tested ones were used soy, olive and vegetable oil, respectively. However, the type of the oil used was not also effective in mercury content.

 Canned tunas made with Longtail tuna (*tongol tuna*) and yellowfin tuna (Albacore tuna) of the Persian Gulf were studied in this paper. Hence, other fish species such as bigeye tuna and Skipja tuna could accumulate different levels of mercury in their tissues. In all 10 brands tested for the mercury content, its concentration was lower than the standard one. Besides, only 7.5% of canned tuna samples, which was 4% lower than 2009 study, had more than 300 ppm mercury.

**Table 1.** The recovery rate of different mercury concentration spikes in a fixed weight of canned tuna samples.

**Sample No.**	**Weight (g)**	**Spiked mercury concentration (ppb)**	**Recovered mercury concentration (ppb)**	**Recovery Percent**
8	0.8	2	0.14 ± 1.88	94
8	0.8	5	5.12 ± 0.18	102
8	0.8	10	10.33 ± 0.23	103
8	0.8	20	0.34±19.71	98

**Table 2:** Ranges and averages of mercury and methyl mercury contents of 10 canned tuna brands

**Brand ** **No**	**Mercury range (ppb)**	**Mercury average (ppb)**	**Methyl mercury range (ppb)**	**Methyl mercury average (ppb)**	**Methyl mercury to mercury content ratio (%)**
1	257.9-120.6	189.3	227.3-96.4	160.4	84.7
2	116.1-75.6	95.9	91.7-57.5	74.6	77.8
3	206.1-88.6	147.3	162.8-70.9	116.9	79.4
4	154.4-143.4	148.9	123.5-119.1	121.3	81.5
5	315.2-185.7	233.1	267.9-139.2	188	80.7
6	266.8-228.2	247.9	221.5-184.8	202.2	81.6
7	294.7-252.7	273.8	235.8-199.6	217.7	79.5
8	227.1-117.6	166.1	197.8-92.6	135.4	81.5
9	182.6-145.3	182.6	146.1-122.2	146.1	80
10	177.4-79.1	76.3	143.7-63.3	60.7	79.6
Total average		177.5		143.7	80.6

**Table 3:** **. **Comparing mercury and methyl mercury contents of canned tuna of the Persian Gulf in different years

**Year**	**Mercury average ± SD**	**Methyl mercury average ± SD**	**Methyl mercury to mercury ratio (%)**	**Total range**	**Reference**
2004	117 ± 57.5	-	-	43-253	(38)
2005	146.6 ± 63.3	-	-	80.5-289.5	(24)
2009	211.6 ± 25.4	173 ± 22.4	80	110-355	(20)
2015	177.5 ± 58.8	143.7 ± 50.7	80.6	57.5-315.2	Current study

 FDA sets 1 ppm as the allowed content of mercury while EPA sets it on 0.5 ppm. This limit is 0.5 ppm in Japan and less than 0.3 ppm for human consumption in Japan (33). These differences in the allowed mercury concentration make the consumers confused. Many countries have set more conservative standards to assure the health of food products (5). Some believe that the practical level should be decreased to 0.185 ppm to preserve endangered population while products with higher content of mercury should be labeled (34). In some reports, the mercury content exceeded the allowed one. The average mercury content in 39 canned tuna of 5 different brands were reported 0.65 in Brazil while the content was higher than 0.5 and1 ppm in 51% and 15% of the samples, respectively (35). In another study on 3 different brands, it was reported that 5% of the samples had mercury contents higher than allowed content defined by FDA (36). The US has announced 0.456 ppm as the total mercury of the canned products by analyzing 168 samples. The mercury content of 25% of the samples was higher than the standard level (0.5 ppm) while the maximum level achieved was 0.956 ppm (5). Researcher's observations about the canned tunas sent to Toxicology Research Center showed the increased mercury content in tested samples to the extent that even some samples had up to 1.5 ppm total mercury. It is necessary to mention that various studies reported significantly different average content of mercury in canned tunas. However, there was no difference among the mercury contents of different species in most of themand this made the analysis difficult. Fifty canned tunas were tested in the Mediterranean and the average mercury content reported to be 0.29 ppm (37). This average was reported to be 211.6 in tunas of the Persian Gulf (20). Based on the above-mentioned results, it could be concluded that the mercury content varies with the location of fishing and also the species of tuna fish.

## Conclusion

 An increasing trend in the mercury content of Tuna fish could be noticed by analyzing the information provided during the last decade. This trend seems to be logical due to the increment in industrial units of the region and gradual rise of using this element in the recent years. On the other hand, the mercury content of canned tunas of the Persian Gulf decreased in 2009 with different locations of fishing, uncontrolled fishing, diets, fish migration, differences in age and size and so forth as the possible causes. However, a common method was not measuring the mercury content and more information is needed to adopt the information in one system and to achieve to one unique result. The mercury contents measured in this study were also acceptable and lower than the international standard so it could be concluded that consumed canned tunas of the Persian Gulf is not a significant public health concern. However, due to serious dangers of mercury poisoning, especially among the pregnant and the kids, and high consumption of canned tuna, continuous monitoring of the canned tunas with a high number of samples is a necessity even with results of periodic studies in recent years.
